# Biodegradable Magnesium Alloys Developed as Bone Repair Materials: A Review

**DOI:** 10.1155/2018/9216314

**Published:** 2018-03-13

**Authors:** Chen Liu, Zheng Ren, Yongdong Xu, Song Pang, Xinbing Zhao, Ying Zhao

**Affiliations:** ^1^Department of Materials Science and Engineering, Zhejiang University, Hangzhou, China; ^2^Ningbo Branch of China Academy of Ordnance Science, Ningbo, China; ^3^Shenzhen Institutes of Advanced Technology, Chinese Academy of Sciences, Shenzhen, China

## Abstract

Bone repair materials are rapidly becoming a hot topic in the field of biomedical materials due to being an important means of repairing human bony deficiencies and replacing hard tissue. Magnesium (Mg) alloys are potentially biocompatible, osteoconductive, and biodegradable metallic materials that can be used in bone repair due to their in situ degradation in the body, mechanical properties similar to those of bones, and ability to positively stimulate the formation of new bones. However, rapid degradation of these materials in physiological environments may lead to gas cavities, hemolysis, and osteolysis and thus, hinder their clinical orthopedic applications. This paper reviews recent work on the use of Mg alloy implants in bone repair. Research to date on alloy design, surface modification, and biological performance of Mg alloys is comprehensively summarized. Future challenges for and developments in biomedical Mg alloys for use in bone repair are also discussed.

## 1. Introduction

As the largest dynamic biological tissue in the body, bones are composed of inorganic minerals and metabolically active cells surrounded by a large volume of extracellular matrix, and they form a rigid framework that has an irreplaceable role in maintaining life activities, including supporting the body and protecting visceral organs [1, 2]. Surgical treatment of bone injuries has become common, where there are millions of bone injury patients in emergency departments worldwide each year due to involvement in vigorous athletic activities, social instability, traffic accidents, and prolonged human lifespan [3–5]. Bone defects, mainly induced by traumatic avulsions, sequelae of infection-induced bony sequestration, congenital malformations, or neoplastic resections, confront us with an extreme challenge for reconstructive surgery. The need to induce bone regeneration to repair structural bone deficiencies has inspired research on and development of a vast number of bone repair materials [2, 6].

Bone repair is a physiological process influenced by a variety of biomechanical, biochemical, cellular, hormonal, and pathological factors. Continuous bone deposition, resorption, and remodeling and sufficient blood supply promote bone repair [7]. Based on the basic principles of bone tissue healing, different bone repair materials have been developed. For a long time, autograft bones have been considered the gold standard of bone repair materials when replacing damaged or lost bones because they have all the characteristics necessary to stimulate new bone growth of osteoconductivity, osteogenicity, and osteoinductivity. However, resources for these autografts are scarce and secondary surgeries increase the pain experienced by patients. Furthermore, donor-site complications can occur, clinical benefits are not guaranteed, and there is a high rate of associated complications [4, 8, 9]. A large number of alternative bone repair materials have been increasingly used to replace autograft bones and are commercially available as bone substitutes. The most commonly used products are composed of calcium (Ca) phosphate ceramics, Ca sulfate, bioactive glass, natural materials, and biological/synthetic composites [10–15]. However, the clinical performance of these materials is unsatisfactory. For example, some have poor mechanical properties and display limited osteoinduction in the clinic [16, 17]. Metallic materials are another alternative for use in the repair or replacement of diseased or damaged bone tissue. Metallic materials currently widely used in orthopedics include stainless steel and titanium alloys because they are mechanically strong and resistant to fracture [18–21]. However, there is a potential for the release of metallic ions and/or particles through corrosion and/or wear that trigger inflammatory responses that can reduce biocompatibility and lead to tissue loss. Furthermore, the elastic moduli and tensile strength of metals and bone are significantly different, which can cause stress shielding and result in weakening of surrounding bone. These inert implants also often need to be removed via invasive secondary surgeries once the bone fracture has completely healed. To minimize trauma to the patients and decrease medical costs, biodegradable implants could be used to replace traditional metal implants and remove the need for secondary surgeries [22–26].

Magnesium (Mg) alloys have a reputation for being revolutionary biodegradable metal materials in orthopedic applications due to their good biocompatibility, biodegradability, and acceptable mechanical properties [27–30]. The fourth most plentiful cation in the human body, Mg is an element essential in many metabolic processes and is primarily stored in bone tissue. Mg is taken into the body daily in substantial amounts, stimulates the growth of bone cells, and accelerates the healing of bone tissue. Mg alloys are degraded* in vivo *due to the presence of Cl^−^ in the physiological environment, thereby eliminating the need for secondary surgeries to remove the implant. Mg^2+^, a corrosion product of Mg alloy implants, does not cause unexpected complications because excessive Mg cations are easily eliminated in the urine [31–34]. Moreover, Mg alloys have mechanical properties similar to those of bone. Mg alloys are lightweight with densities (1.7–1.9 g/cm^3^) very similar to those of human cortical bone (1.75 g/cm^3^), unlike titanium alloys (Ti-6Al-4V 4.47 g/cm^3^) and stainless steel (about 7.8 g/cm^3^). The elastic modulus of Mg alloys, about 45 GPa, is relatively close to that of natural bone, 3–20 GPa, compared to the elastic moduli of titanium alloys and stainless steel (110 and 200 GPa, resp.). Therefore, the stress shielding from the notable mechanical mismatch between natural bone and metal implants should be mitigated [35–37]. Therefore, Mg alloys are expected to become biocompatible, biodegradable, lightweight, and load-bearing orthopedic implants [22, 38–40].

While research on Mg alloys as bone implants has led to significant progress over the past 20 years, rapid degradation of these materials inside the human body is still a major obstacle hampering their use in the clinic. As biodegradable materials, it is important that the rate of implant degradation matches the rate of healing of the bone tissue, which generally consists of an early inflammatory stage lasting from 3 to 7 days, a reparative stage that leads to a strong healing union lasting about 3-4 months, and then a remodeling phase that can last months to years [41–43]. Therefore, it is necessary for the implant to remain stable for at least 12 weeks [22]. However, the currently available Mg alloys degrade too quickly to hold well during implantation. This fast degradation results in the formation of hydrogen gas cavities, rapid loss of mechanical integrity of the implants, and adverse host tissue reactions, such as local swelling and significant pain within the first week after surgery [44–46].

There have been a number of recent opportunities and challenges in the development of Mg alloys for use in bone repair. Therefore, it is necessary to summarize the findings of the researchers in this field. Compared to recently published reviews [27, 47–53], this paper is more targeted and specifically discusses biodegradable Mg alloys to be used in bone repair. We review the alloying design, surface modifications, and the* in vitro* and* in vivo* biological performance of Mg in bone repair. Novel insights that have been used to improve the compatibility and reliability of biomedical Mg alloys in the bone reconstruction field are also discussed.

## 2. Alloying Design of Magnesium Alloys

Adequate strength, ductility, fatigue resistance, and biocorrosion resistance are important characteristics for biodegradable implants to be used in orthopedic applications. Because adding alloying elements can improve mechanical properties and decrease the corrosion rate of Mg by modifying the structure and phase distribution, several Mg alloys have been designed to meet the requirements of bone repair implant materials [30, 32, 60].

### 2.1. Alloying Elements

Careful selection of alloying elements is the first step in designing Mg alloys. To strengthen Mg-based materials, adding elements such as Al, Zn, Ca, Ag, Ce, and Th can generate different microstructures and improve the mechanical properties of the resulting Mg alloy [71–74]. In terms of corrosion, alloying elements that have electrochemical potentials similar to that of Mg (−2.37 V), such as Y (−2.37 V), Nd (−2.43 V), and Ce (−2.48 V), and have relatively high solid solubility in Mg, such as Sc (25.9 wt.% limit), Gd (23.5 wt.% limit), and Dy (25.3 wt.% limit), can enhance the corrosion resistance by reducing internal galvanic corrosion in physiological environments [37, 75, 76]. Biocompatibility also needs to be considered. Previous reports have shown that biological nutrients (e.g., Ca, Sr, Zn, Si, and Mn) and trace nontoxic elements (e.g., Zr, Nd, and Y) added either independently or together to the Mg matrix do not cause detrimental local tissue responses and can be easily absorbed by surrounding tissues [29, 30, 35, 77–80]. With the development of biodegradable Mg alloys, researchers have started trying to endow Mg alloys with new biomedical functions through alloying. Ca, Sr, Ag, and Cu as biofunctional trace metallic elements have been confirmed to promote bone cell activation and stimulate new bone formation. In addition to promoting osteogenesis, these elements also inhibit bacterial infection after implantation, thereby effectively decreasing morbidity and mortality, by making the environment alkaline and releasing antimicrobial metallic ions [81–86].

### 2.2. Alloy Systems

Due to having a combination of good mechanical properties and corrosion resistance, some commercial Mg alloy systems have been selected as biodegradable Mg alloys at an early stage. Commercial Mg alloys used in biological research include the AZ (Mg-Al-Zn), WE (Mg-RE-Zr), and ZK (Mg-Zn-Zr) series alloys.

AZ series alloys, particularly AZ31 (Mg-3Al-1Zn) and AZ91 (Mg-9Al-1Zn) alloys, have been extensively studied both* in vitro* and* in vivo* in recent years [46, 87–89]. It has been reported that AZ31 and AZ91 alloys release hydrogen upon degradation in physiological environments, leading to a significant increase in both pH and Mg ion concentration [90]. In Hank's solution, the AZ31 alloy degrades more slowly than the AZ91 alloy, but there is no significant difference* in vivo* [91, 92]. Short-term* in vivo* studies of AZ31 and AZ91 alloys have also revealed that a biocompatible Ca phosphate protective film layer covers their surfaces and increases the formation of new bone mass around the implants [92, 93].

WE series alloys have good biocorrosion resistance because they form a rare-earth (RE) oxide film in aqueous environments. It has been reported that WE54 (1.58 Nd, 4.85 Y, 0.28 Zr, 0.08 Ce, 0.13 Gd, 0.16 Er, 0.13 Yb, and balanced Mg in wt.%) has marginally higher resistance to degradation* in vitro* than pure Mg and heat treatment impacts its degradation [94]. Witte et al. analyzed the* in vivo* degradation of four different Mg alloys and confirmed that WE43 (4.16 Y, 3.80 RE, 0.36 Zr, 0.20 Zn, and 0.13 Mn, all in wt.%) has good biocompatibility [93]. However, an increase in Al ion concentration in the brain is associated with the occurrence of Alzheimer's disease and severe hepatotoxicity has occurred after the administration of RE elements, such as Y, Ce, and Pr [6].

Recently, ZK series alloys, especially ZK40 (Mg-4Zn-0.5Zr) and ZK60 (Mg-6Zn-0.5Zr), have attracted the attention of researchers because of the good biocompatibility of the component elements [95–97]. A daily intake of 11 mg Zn and 50* μ*g Zr is permissible, so Mg-Zn-Zr alloys are more attractive than Mg-Al-Zn and Mg-RE-Zr alloys in terms of element biocompatibility and biosafety and are candidate biodegradable metals for use in bone repair devices [25]. However, the extremely high rates of degradation of Mg-Zn-Zr alloys are alarming and restrict their future development.

In addition to the above commercial Mg alloy systems, new Mg alloys have also been developed for use in orthopedic applications, including Mg-Ca, Mg-Sr, Mg-Zn, and Mg-RE alloy systems.

Ca, acting as a grain-refining agent in Mg alloys, can stabilize grain size at levels up to 0.5% of the Ca content and cause slight decreases with further addition [98]. As a major component of human bone, Ca is essential for bone cell signaling and beneficial to bone healing. It has been reported that Mg-1Ca alloy does not induce cytotoxicity and osteoblasts and osteocytes are highly active around Mg-1Ca alloy pins implanted in rabbit femoral shafts, thus demonstrating good biocompatibility and bioactivity [84].

Strontium (Sr) and Ca belong to the same family and have similar physical and chemical properties and biological functions. Brar et al. studied Mg-*x* wt.% Sr (*x* = 0.5, 1.0, and 1.5 wt.%) alloys and found that the Mg-0.5Sr alloy degraded the slowest [35]. Zhao et al. and Gu et al., respectively, reported that the as-extruded Mg-0.5Sr and as-rolled Mg-2Sr alloys had the best combination of corrosion resistance, high strength, and* in vivo* biocompatibility [86, 99].

Zinc (Zn) is one of the most abundant essential nutrients in the human body and is safe for use in biomedical applications [100]. The rate of Mg corrosion can be reduced by increasing the mass fraction of Zn mixed with Mg, thus strengthening the mechanical properties of Mg through solid solution hardening [101]. Cai et al. reported that a Zn content of up to 5 wt.% in Mg-Zn binary alloys exhibits grain boundary, solid solution, and secondary phase strengthening, resulting in improved resistance to corrosion and mechanical properties [102]. Mg-6Zn alloy has good biocompatibility* in vitro *based on hemolysis and MC3T3-E1 cell adhesion assays [103].

Because Mg-RE alloys have good mechanical properties and corrosion resistance, new Mg-RE alloys, such as Mg-Y, Mg-Nd, Mg-Gd, Mg-Ce, and Mg-Ld, have been studied. Among these, Mg-Nd alloy has a much slower corrosion rate than the other alloys [74]. Mg-Y alloy was prepared using a zone solidification method and improved corrosion resistance and mechanical properties [104]. Mg-Y-Zn alloy contains an interesting combination of preferred microstructural, mechanical, electrochemical, and biological properties, making it very promising for use as a biodegradable implant material [105].

### 2.3. Alloy Microstructures

Alloying elements in Mg alloys may exist in the form of second-phase particles and precipitate in grains or grain boundaries, substantially enhancing mechanical properties through second-phase strengthening.

**Figure 1:**
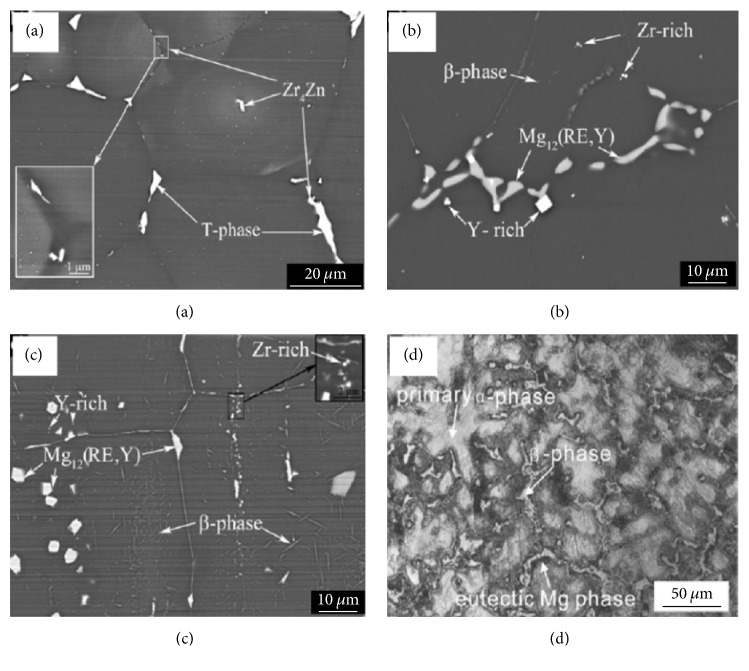
Typical morphologies of second phases in (a) as-cast ZE41, (b) as-cast WE43, (c) as-forged WE43 [54], and (d) AZ91D alloys [55].

Figure 1 presents the typical morphologies of second phases for Mg alloys and Table 1 presents the second phases of biodegradable Mg alloys. Compared to Mg matrix, second phases have higher potentials and may facilitate corrosion, leaching into the physiological environment accompanied with the degradation of the matrix. Kannan investigated the degradability of Mg_17_Al_12_ phase in simulated body fluid (SBF) using electrochemical measurements and found that the degradation rate of Mg_17_Al_12_ was lower than that of bare Mg. Our previous study demonstrated that pitting corrosion occurs with crackings for Mg_17_Al_12_ phase in Hank's solution and degrades much slower than AZ31 alloy and pure Mg [106].

When assessing Mg alloy implants for use in bone repair, the stability of second phases and Mg matrix under different conditions may have significantly influenced degradation and biological responses to the implant in the body. Yang et al. theoretically investigated the thermodynamic stability of four conventional second phases for Mg-Zn-Zr, Mg-Ca, Mg-Sr, and Mg-Al-Zn alloys, as well as Mg matrix in bioabsorbable Mg alloys, via the Dmol^3^ calculation method. The second phases had higher phase stability than Mg matrix, but the phase stability was quite different for different types of second phases and second-phase-4H_2_O systems [71]. In order to evaluate the effect of second phases on the biological safety of biodegradable Mg alloy implants, Mg_17_Al_12_ second phase from Mg-Al-Zn alloys was investigated for* in vitro* biocompatibility and phagocytosis by macrophages. Mg_17_Al_12_ second phase did not induce hemolysis and had excellent cytocompatibility. Mg_17_Al_12_ particles are processed in endolysosomal compartments and lysosomes play a major role in digesting Mg_17_Al_12_ particles [107].

However, not all the alloying elements in Mg alloys form second-phase particles. As mentioned above, some alloy elements have relatively high solid solubility in Mg, such as Y (12 wt.% limit), Sc (25.9 wt.% limit), Gd (23.5 wt.% limit), and Dy (25.3 wt.% limit), and can exist in the form of solid solutions, thus achieving solid solution strengthening. In the solution, the original crystal structure of magnesium remains unchanged, but a lattice distortion is produced and thus the motion of dislocations becomes impeded, which leads to the enhancement of strength of Mg. Gao et al. explored the effects of solid solutions on the mechanical behavior of binary Mg-Y single-phase alloys. They found enhanced hardness as the Y content increased at room temperature because of large differences in the atomic radii of Y and Mg and a relatively wide range of solubilities [108]. Moreover, solid solution alloying also potentially affects degradation of Mg alloys by improving corrosion resistance by reducing internal galvanic corrosion between the second phase and Mg matrix. Zhang et al. studied the effect of solid solution treatment on the corrosion and electrochemical behaviors of Mg-15Y alloy and found that solution treatment decreased the extent of galvanic corrosion due to the dissolution of Mg_24_Y_5_ second phase into the matrix [109]. Therefore, solid solution might be a feasible alternative for generating a single-phase Mg alloy and can help improve the corrosion resistance of Mg alloys in orthopedic applications.

### 2.4. Impurities in Magnesium Alloys

During casting and refining, magnesium always introduces superfluous amounts of impurity elements. Impurity elements in Mg alloys usually include iron (Fe), nickel (Ni), and copper (Cu) [66, 110]. These elements can significantly accelerate Mg corrosion when their concentrations exceed the limits of tolerance [111–113]. Standards for Mg impurity elements are 35–50 ppm for Fe, 20–50 ppm for Ni, and 100–300 ppm for Cu (wt.%). Below the tolerance limits, no impurity particles are formed and, thus, no electrochemically active cathodic sites exist to accelerate corrosive attack, which keeps the corrosion rate very slow. When levels are above the tolerance limits, Fe, Ni, and Cu in Mg alloys significantly increase the corrosion rate due to the low solubility of these elements and their distinctly more noble position in the electrochemical series [66]. Atrens et al. found that impurity elements notably accelerate salt-water corrosion of Mg binary alloys [114]. Recent studies have shown that adding silicon (Si) to the reactive impurity elements Fe, Ni, and Cu is detrimental to corrosion, as it plays a critical role in promoting the formation and growth of Fe-rich particles. Lee et al. suggested that corrosion of Mg is dependent on the content ratio of impurities, such as the Fe/Mn ratio, rather than their absolute content. As the Fe/Mn ratio increases, the high rate of corrosion stage extends [5]. In addition to accelerating corrosion, excessive impurity elements are also harmful to biocompatibility. For example, Ni leaching into the body has toxic biological effects and high levels of Cu exert a toxic effect at cell surfaces [115]. In order to reduce impurity during casting and refining, the crucible, stirrer, and mold containing no such elements are prudently utilized [31].

**Figure 2:**
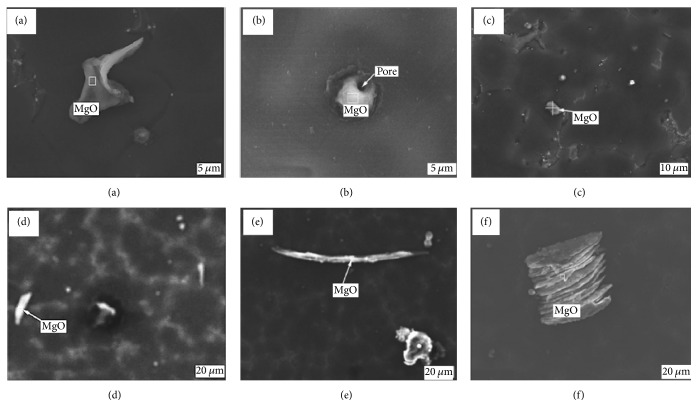
Scanning electron microscope (SEM) images of MgO inclusions in Mg-Gd-Y-Zr: (a) Z-shaped, (b) spherical, (c) block, (d) rod-like, (e) needle-like, and (f) lamellar MgO [56].

As the chemical properties of Mg alloys are very active, a large amount of nonmetallic inclusions is also produced during casting and refining which act as additional major impurities in Mg alloys [116]. The main nonmetallic inclusions include MgO, Mg_3_N_2_, MgF_2_, MgS_2_, and AlF_3_. These nonmetallic impurities primarily come from the oxidation of Mg alloys in ambient atmospheres. For example, MgO, a common Mg alloy inclusion, is produced when Mg and O_2_ react in the air.  Figure 2 illustrates the different morphologies of MgO impurities in Mg-Gd-Y-Zr alloy [56]. Mg_3_N_2_ is attributed to Mg and N_2_ combining in the air. When Mg alloys smelt under the protection of SF_6_ gas, MgF_2_ and MgS inclusions may form from reactions between SF_6_ and liquid Mg. As the nonmetallic impurities significantly reduce the castability, mechanical properties, and corrosion resistance of Mg alloys, purification technology is undergoing continuous development [117]. The common methods of purifying Mg alloys include gas purge, flux purification, filtering purification, RE purification, and electromagnetic purification methods [116].

## 3. Surface Modifications of Magnesium Alloys

In order to efficiently improve the corrosion resistance of Mg alloys in physiological environments, as well as maintain their mechanical integrity and ameliorate interfacial biocompatibility, various surface modifications have been developed. Distinct from alloying techniques, surface modifications directly insulate Mg alloys from the surrounding biological environment and prevent the penetration of body fluid into substrates [100, 118, 119]. Based on whether a new phase is generated on the surface of the Mg alloys, the methods of surface modification can be classified into three categories: chemical modifications, physical modifications, and a combination of these two methods [120].

### 3.1. Chemical Modifications

Chemical modifications are defined as new phases covering the surface of Mg alloys that are synthesized through chemical or electrochemical reactions. This method removes the native oxide layer that has fewer passive properties, such as an inability to efficiently protect against corrosion, but forms easily due to the high reactivity of Mg matrix. Chemical modifications generally include acid etching, alkaline heat treatment, fluoride treatment, anodic oxidation, and microarc oxidation (MAO) [120].

Acid etching is a pretreatment method commonly used to remove the coarse scale produced during manufacturing and replace the native oxide layer with a more compact passivated layer [121]. Turhan et al. reported that acid etching with a 2.5% H_2_SO_4_ solution greatly enhances the resistance of AZ91D alloys to degradation [122]. In addition, alkaline heat treatment, a simple and economical method, creates a Mg(OH)_2_ barrier layer on substrate surface that slows down the corrosion rate of Mg alloy [123]. It has been reported that the corrosion rate of Mg is decreased through NaOH treatment, where an NaOH concentration of 1 M leads to the slowest corrosion rate, through the formation of a protective layer [123, 124]. Fluoride treatment of Mg alloys replaces the original oxide film with a thin and more homogeneous MgF_2_ layer with higher polarization resistance. The advantages of the MgF_2_ layer include a high density, low water solubility, and nontoxicity when fluorine ions are released into the host organism. Witte found out that MgF_2_ coating slows* in vivo *corrosion of LAE442 alloy without observably elevating fluoride concentrations in the adjacent bone [125]. Moreover, fluoride can stimulate osteoblast proliferation, increase new mineral deposition in cancellous bones, and decrease the solubility of bone tissue upon incorporation into the bone [88]. An experimental study in dogs found that fluoride-modified implant surfaces promote osteointegration during the early phase of healing following installation of the implant [126].

**Figure 3:**
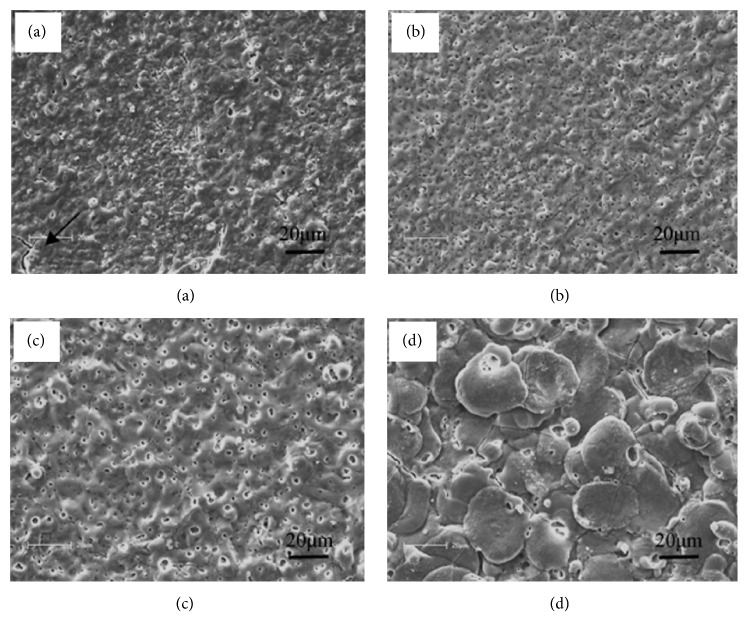
Surface morphologies of ZK60 alloy coated by microarc oxidation at voltages of (a) 230 V, (b) 300 V, (c) 370 V, and (d) 450 V [29]. Some microcracks can be found on the 230 V coating, as marked by the black arrow in (a).

Anodic oxidation is an electrochemical process that produces a thick and stable oxide film on metals. Lei et al. created an Mg oxide film on AZ31B Mg alloy by anodic oxidation at a constant current. This film efficiently delays degradation of AZ31B Mg alloy without having any adverse effects on osteoblast proliferation or new bone formation [127]. MAO is a high-voltage plasma-assisted anodic oxidation process that is widely employed to modify the surface of biodegradable Mg alloys. MAO coatings are very hard and have good wear resistance, moderate corrosion resistance, and better thermal stability and dielectric properties [128]. Lin et al. prepared forsterite-containing MAO coatings on ZK60 Mg alloy to slow down degradation and improve the biological properties of the alloy. It was found that the resistance to corrosion from the MAO coating increased as the preparation voltage increased. Compared to bare ZK60 Mg alloy, MAO-coated ZK60 has a dramatically lower hemolytic ratio and no cytotoxicity to L929 cells.  Figure 3 presents the surface morphologies of ZK60 alloy with MAO coatings generated at different voltages [29].

### 3.2. Physical Modifications

Different from the chemical methods, no chemical bonds were formed between the surface and the substrates for physical modifications. The modifications aim to offer a physical barrier to improve the corrosion resistance of magnesium substrates. The physical modifications can be performed by introducing apatite coatings, polymer coatings, laser surface processing, or cold spray coatings [120, 129].

Apatite is a main inorganic component of natural bone. It can remarkably promote the recovery of bone fracture due to its excellent bioactivity. Besides, apatite also could improve the degradation resistance of implants as a protective layer due to its relatively low solubility and high thermal stability [130].

As one important member of the apatite family, hydroxyapatite (HA) shows the closest chemical composition with bone mineral and is widely used to coat magnesium alloys for bone repair [120]. Wang et al. developed an HA coating on ZK60 Mg alloy with HA and found that it prevented the degradation of the alloy and increased cytocompatibility for L929 cells, rendering ZK60 alloy more suitable for orthopedic applications. In addition, no significant deterioration in compression strength was noted in the coated alloy compared to the uncoated one [131].

Polymer coatings are also promising Mg alloy modifications for use in orthopedic applications. Gray-Munro et al. explored the influence of polymer coating on the corrosion rate of AZ31 Mg alloy in SBF using PLA, which is a semicrystalline biodegradable polymer, and found that the coating prevented corrosion, especially during the early stages of implantation [90].

Laser surface processing, which uses a high-energy laser beam, has also been employed to regulate biodegradation of Mg alloys and has been found to cause secondary phase dissolution and create a fine grained structure. Coy et al. found significant dissolution of the second phase of Mg_17_Al_12_ in AZ91D when using laser surface processing [132]. Similar results were reported by Guo et al. and Khalfaui et al. for WE43 and ZE41 alloys using laser processing [133, 134]. Appreciable improvements in resistance to corrosion have also been observed for the aforementioned modified alloys [135].

Cold spray technology is a viable method for surface engineering of Mg alloys. The deposition of cold spray coatings involves ballistic impingement of particles, usually ranging in size from 1 to 100* μ*m, accelerated by a high-velocity gas stream and sprayed towards the substrate surface. A low temperature process, cold spray is particularly suitable for the deposition of bioactive coatings on Mg alloys, making it possible to depress oxidation and phase transformation of the substrate. Noorakma et al. recently studied the deposition of HA on an AZ51 alloy using a modified cold spray process and found that this modification helped retain the characteristics of HA. Immersion in SBF for up to 14 days revealed that HA-coated AZ51 alloy was bioactive and facilitated apatite formation [136].

### 3.3. Chemical and Physical Modifications

**Figure 4:**
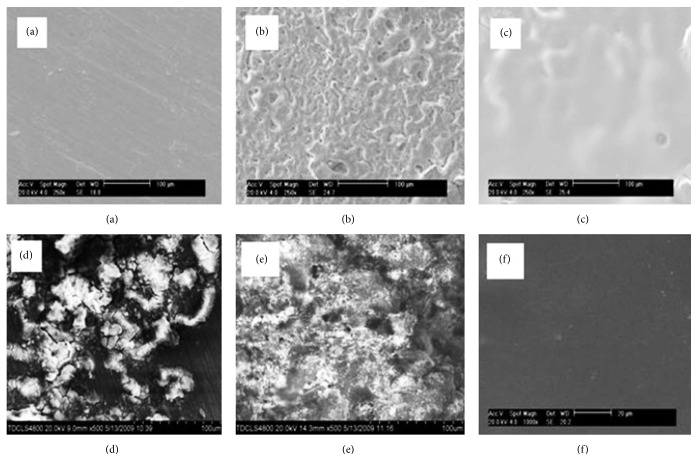
SEM images of sample surface morphology before (a) WE42, (b) WE42-MAO, and (c) WE42-MAO/PLLA and after (d) WE42, (e) WE42-MAO, and (f) WE42-MAO/PLLA were submerged in Hank's solution at 37°C (pH = 7.4) [57].

Considering the limitations of single chemical and physical treatments, composite modifications that involve both chemical and physical treatments have been gaining increasing attention. It has been reported that double-modified layers effectively improve biodegradation resistance of substrates and control degradation rates over a larger range [120]. Guo et al. fabricated an MAO/poly-L-lactic acid (PLLA) composite coating on WE42 alloy surfaces by sealing PLLA to the MAO coating through physical interlocking. This MAO/PLLA-modified WE42 alloy was found to have good corrosion resistance and cytocompatibility.  Figure 4 presents the surface morphologies of WE42, WE42-MAO, and WE42-MAO/PLLA before and after being submerged in Hank's solution for four days [57]. As shown in Figures 4(a) and 4(d), WE42 Mg alloy was severely corroded by Hank's solution. The surface of the WE42 experienced strong corrosion as shown in Figure 4(d) based on deeper and wider cracks and holes, as well as the deposition of white flocculent accumulations. Micropores and microcracks were randomly distributed on the surface of the MAO coating (Figure 4(b)). After submersion, the MAO coating was corroded with little white flocculent deposits on the surface (Figure 4(e)). The biocompatible PLLA sealing layer was smooth and uniform, overlaying cracks and pores on the surface of the MAO coating (Figure 4(c)). As shown in Figure 4(f), there were no notable changes to the surface of the MAO/PLLA, where the surface of the WE42-MAO/PLLA sample remained covered with an intact layer that displayed no signs of corrosion.

## 4. Biological Performance of Biodegradable Magnesium Alloys as Bone Implants

It is critical for biodegradable Mg alloys to have good biocompatibility in the body in order to be used in the clinic [130]. Therefore, the* in vitro *and* in vivo* biological performance of biodegradable Mg alloys has been examined for many years [137].

### 4.1. *In Vitro* Biological Performance

**Figure 5:**
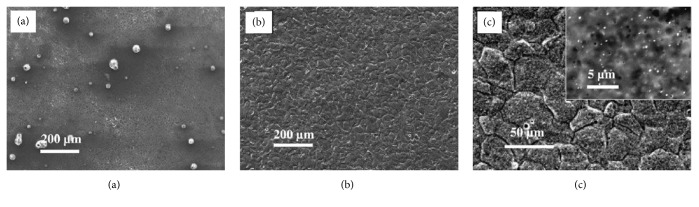
Surface morphology of M1A after soaking in A-SBF for 30 min: (a) original surface, (b) surface after cleaning, and (c) high-magnification view of surface after cleaning [58].

*In vitro *experiments can be used to simulate and predict corrosion and biocompatibility of Mg alloys* in vivo *[138]. Compared to* in vivo* experiments,* in vitro* experiments are more convenient and can provide quick and reasonable feedback concerning efficacy [139]. Gu et al. studied the* in vitro* corrosion and biocompatibility of nine binary Mg-1X (wt.%, X = Al, Ag, In, Mn, Si, Sn, Y, Zn, and Zr) alloys using SEM, X-ray diffraction, tensile tests, immersion tests, electrochemical corrosion tests, cell culture, and platelet adhesion. The addition of alloying elements influenced the strength and corrosion resistance of Mg. Al, Si, Sn, Zn, and Zr improved the strength of Mg, while Al, In, Mn, Zn, and Zr slowed down corrosion of as-cast Mg-X alloys in both SBF and Hank's solutions. Conversely, Si and Y negatively impacted Mg corrosion. Cytotoxicity assays indicate that Mg-1Al, Mg-1Sn, and Mg-1Zn alloy extracts do not significant reduce the viability of fibroblasts (L-929 and NIH3T3), Mg-1Al, Mg-1Si, Mg-1Sn, Mg-1Y, Mg-1Zn, and Mg-1Zr alloy extracts do not have significant toxicity against osteoblasts (MC3T3-E1), and Mg-1Al and Mg-1Zn have no negative effects on the viability of blood vessel-related cells (ECV304 and VSMC). In hemolysis assays, Mg-1In, Mg-1Mn, Mg-1Si, and Mg-1Y alloys had low ratios of hemolysis of less than 5%. Adhered platelets are approximately round in shape and have slight spreading of pseudopodia, but fewer were adhered for alloys compared to the pure Mg control [140]. Wang et al. investigated* in vitro* cellular responses and degradation of the Mg alloy M1A (Mg-1.42 wt.% Mn) in SBF and albumin-containing SBF (A-SBF, 40 g/L). They found that the corrosion of M1A was strongly affected by the presence of albumin due to the synergistic effects of albumin adsorption and chelation. M1A samples had well-spread cells and good cell viability, implying that M1A Mg alloy has the potential to serve in biodegradable implants.  Figure 5 presents the surface morphology of M1A after soaking in A-SBF for 30 min [58].  Figure 5(a) suggests that the presence of albumin does not significantly influence the formation of the passivation layer within the first 0.5 h of immersion. However, assessments of the surface after cleaning (Figures 5(b) and 5(c)) reveal that the grain boundaries are still the preferred sites for initiation of corrosion and the corrosion was relatively uniform across the test surface. However,* in vitro *assays cannot completely recapitulate* in vivo* experiments because* in vivo* environments are more complex [141]. Witte et al. investigated the effects of* in vitro* and* in vivo* corrosive environments on the corrosion rates of gravity-casted AZ91D and LAE442 Mg alloys and found that corrosion was about four orders of magnitude slower* in vivo* than* in vitro* [92].

### 4.2. *In Vivo* Biological Performance

*In vivo* animal experiments must be performed to optimally mimic physiological environments of human body prior to clinical experiments.* In vivo* animal experiments help characterize local tissue reactions to Mg-based implants through follow-up testing, including serum analysis, radiographic examination, micro-CT investigations, histology analysis, and implant examination [142]. Local bone responses to biodegradable Mg alloys depend on the rate of degradation, corrosion products, and stability of the Mg alloys.

**Figure 6:**
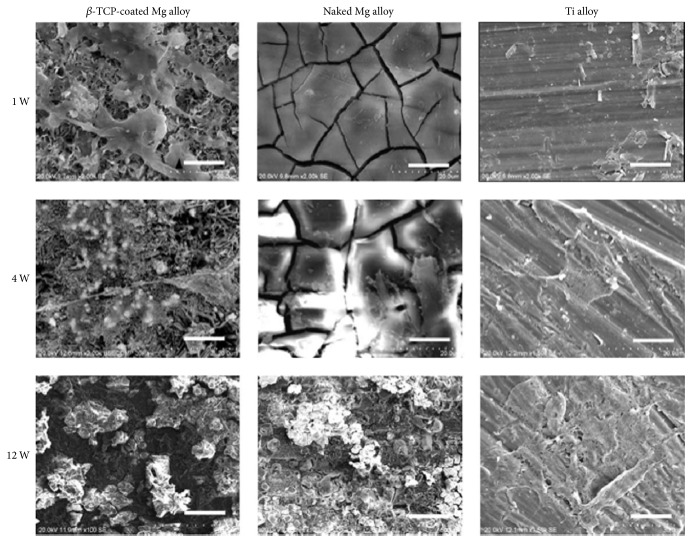
SEM images of *β*-tricalcium phosphate-coated AZ31, naked AZ31, and Ti-6Al-4V alloy rod samples after implantation for 1, 4, and 12 weeks. Scale bar = 5 *μ*m [59].

Zhang et al. implanted Mg-Zn-Mn alloy into rats to investigate the* in vivo* degradation of Mg alloy, response of the bone to the biodegradable Mg implant, and effect of the degradation of Mg alloy on blood composition and organs. Mg-Zn-Mn alloy was found to degrade at different rates in the marrow cavity and cortical bone. New bone tissue, but not fibrous capsule, formed around the Mg implants 6 weeks after implantation. More new bone tissue, as well as membrane, was found around the implant 10 and 26 weeks after implantation. The degradation of the Mg-Zn-Mn implant caused little change to the blood composition, liver, and kidneys [143]. Dziuba et al. developed a new degradable Mg alloy, ZEK100, and explored its long-term degradation and biocompatibility in adult female New Zealand white rabbits. Importantly, ZEK100 degrades slowly* in vivo*. However, favorable* in vivo* degradation is not necessarily associated with good biocompatibility and the absence of general pathological disorders does not definitively indicate that Mg implants have acceptable biocompatibility. In this study, ZEK100 caused various local pathological effects in the form of severe bone alterations [142]. Chai et al. implanted *β*-tricalcium phosphate- (TCP-) coated AZ31 Mg alloys into the femurs of rats after predrilling with 1 mm hand-operated drills to evaluate implant osteogenesis and biodegradability.  Figure 6 shows the SEM of the rod samples of *β*-TCP-coated AZ31, naked AZ31, and Ti-6Al-4V alloys after implantation for 1, 4, and 12 weeks [59]. For the *β*-TCP-coated Mg alloy, cells and cell secretion proteins were found on the surface after the 1st week. After 4 weeks, the rod implant was covered with a large amount of organic proteins. After 12 weeks, degradation products and cracks were thicker on the surface than at the previous timepoint. On the naked Mg alloy, many cracks were clearly seen after 1 week. After 4 weeks, cell secretion proteins were found on the surface. After 12 weeks, a thin excreted matrix layer that almost covered the naked Mg alloy sample was observed. By comparison, the Ti alloy surface morphology was the same at different timepoints. This demonstrates that the *β*-TCP coating slows down degradation of naked Mg alloy at the early stages of implantation and confirms that the *β*-TCP coating greatly improved osteoconductivity and osteogenesis in the early 12-week postoperation period.

## 5. Conclusion and Suggestions

This review presented and discussed recent research and developments on Mg alloy for use in bone repair. Significant efforts have been made to improve the mechanical properties, corrosion resistance, and biocompatibility of Mg alloys through alloying design and surface modification. In summary, there is great potential for the future use of Mg alloys in bone repair as surgical implant materials. Although a vast number of studies have focused on biodegradable Mg alloy implants, which are expected to reduce the need for follow-up surgeries and lead to safer, more effective bone repair, improvements are needed and suggestions for future research are presented in this article.

To better mimic the performance of Mg alloys in physiological environments, targeted animal models need to be created. For example, an ovariectomized rat model was built to explore the effects of 10% SrHA coatings on implant fixation and prophylaxis of postmenopausal osteoporosis [144]. Waselau et al. created triangular fragments with 1 cm long arms using a Y-shaped osteotomy of the second and fourth metatarsal bones in horses and compared the effects of biodegradable Mg phosphate cement, Ca phosphate cement, and no cement on bone repair, biocompatibility, and bone adhesion [145]. The above-described animal models, as well as traditional bone damage models, should be adapted for future studies on the use of Mg alloys for bone repair.

With regard to the feasibility of using biodegradable Mg alloys in bone repair surgery, the interlocking of bone implants, such as nails, screws, needles, and plates, into the surrounding bone must be biomechanically tested. It is important to assess the strength of bone-implant fixation* in vivo* by comparing the implants of interest with commonly used implants. Erdmann et al. compared the biomechanical properties of degradable Mg-0.8Ca alloy and commonly used stainless steel (S316L) screws using uniaxial pull-out tests in an MTS 858 Mini Bionix at a rate of 0.1 mm/s. Mg-0.8Ca had good tolerability and biomechanical properties comparable to S316L during the first 2-3 weeks after implantation. Therefore, its use as a biodegradable implant is conceivable [23]. Castellani et al. investigated the bone-implant interface strength and osseointegration of a novel biodegradable Mg alloy (Mg-Y-Nd-HRE, based on WE43) and compared it to a titanium control (Ti-6Al-7Nb). By comparison, Mg-Y-Nd-HRE alloy not only enhanced the response of the bone but also had excellent interfacial strength, thus fulfilling two critical requirements for use in bone implants [146]. Creating a mechanically stable bone-implant interface is particularly critical to the successful clinical use of bone repair implants. Therefore, additional biomechanical research is required in the future.

Because of the complexity of the physiological environment of the human body, long-term studies are required to investigate* in vivo* degradation and biocompatibility of biodegradable Mg alloys. In addition to the above suggestions, future work should focus on the topics described below. The development of controllable degradation of biodegradable Mg alloys via either novel or traditional strategies, such as processing control and bionic coating, is required. An example is the development of biofunctional alloy systems using human essential nutrients in alloying [81]. In addition, because bone vasculature plays a vital role in bone development, remodeling, and homeostasis, angiogenesis of Mg-based implants should also be a focus of research [147]. In order to obtain more reliable biosafety information and prepare for clinical trials, it is necessary to investigate the longer-term effects of Mg alloy implants on tissues and organs. The* in vivo* performance of biodegradable Mg alloys will likely improve in the near future and, therefore, Mg alloy implants will play more important roles in the treatment of orthopedic diseases.

## Figures and Tables

**Table 1:** Common second phases of select biodegradable Mg alloys.

Biodegradable magnesium alloys	The second phases in magnesium matrix
AZ31B [61], AZ61D [62]	Mg_17_Al_12_
AZ91D [63, 64]	Mg_17_Al_12_, Al_8_Mn_5_
Mg-Ca [65]	Mg_2_Ca
Mg-Sr [4, 65]	Mg_17_Sr_2_, Mg_2_Sr
Mg-Zn [66]	MgZn_2_
Mg-Zn-Ca [67]	Mg_2_Zn_3_
Mg-Si [66]	Mg_2_Si
Mg-Al-Si [68]	Mg_2_Si
WE43 [69]	Mg_24_Y_5_, Mg_41_Nd_5_, Mg_12_Nd
ZK60 [70]	MgZn, MgZn_2_
